# Supplementary material for: Boosting the Photocatalysis of Plasmonic Au-Cu Nanocatalyst by AuCu-TiO2 Interface Derived from O2 Plasma Treatment
Source: Int J Mol Sci. 2023 Jun 22;24(13):10487. doi: 10.3390/ijms241310487 (PMC10342079; doi:10.3390/ijms241310487)
Supplement: Supplementary file 1 [file ijms-24-10487-s001.zip › Supporting Material.pdf]

Supporting Material

Boosting the photocatalysis of plasmonic Au-Cu nanocatalyst by AuCu-TiO_2 interface derived from O_2 plasma treatment

Bin Zhu [1], Xue Li [1], Ye-Cheng Li [2,*], Jing-Lin Liu [1], Xiao-Min Zhang [3,*]

[1] *College of Environmental Sciences and Engineering, Dalian Maritime University, Dalian 116024, China; binzhu@dlmu.edu.cn (B.Z.)*

[2] *Laboratory of Plasma Physical Chemistry, Dalian University of Technology, Dalian 116024, China*

[3] *State Key Laboratory of Catalysis, Dalian National Laboratory for Clean Energy, Dalian Institute of Chemical Physics, Chinese Academy of Sciences, Dalian 116023, China*

* *Corresponding authors. E-mail addresses: liyc_119@mail.dlut.edu (Y.-C. Li), zhangxiaomin@dicp.ac.cn (X.M. Zhang),*

ORCID

Bin Zhu: 0000-0003-0803-4873

Yecheng Li 0000-0001-7583-3961

Jinglin Liu: 0000-0001-7583-3961

Xiaomin Zhang: 0000-0001-5155-6779

The calculation of mass-specific reaction rate

In this work, mass-specific reaction rate for the visible light (VL) photocatalytic oxidation (PCO) of CO was defined as Eq. S1:

$$r_{CO} = \frac{F_{\mathrm{in}} \cdot C_{\mathrm{CO}}^{\mathrm{in}} \cdot X_{\mathrm{CO}}}{m_{\mathrm{Au}}} \tag{S1}$$

where F_{in} is the total flow rate of inlet gas, and $C_{\mathrm{CO}}^{\mathrm{in}}$ denotes the concentrations of CO in inlet gas. X_{CO} is the conversion of CO, and m_{Au} represents the actual mass of Au in the nanocatalysts. During the calculation of mass-specific reaction rate, X_{CO} was controlled under differential reaction conditions (below 18 %).

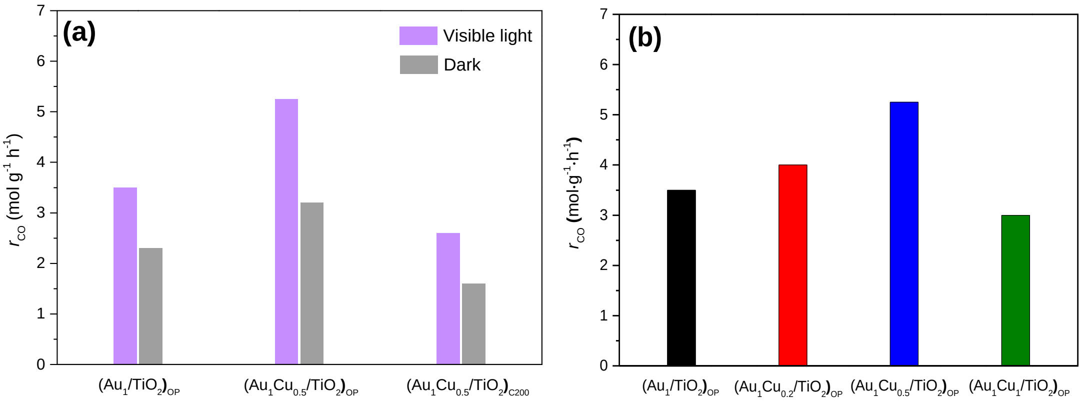

Figure S1 (a) Comparison of mass-specific reaction rates for CO oxidation with and without VL irradiation over different nanocatalysts; (b) VL PCO of CO over Au-Cu/TiO_2 nanocatalysts treated by O_2 plasma with different Cu contents. The $(Au_1Cu_{0.2}/TiO_2)_{OP}$, $(Au_1Cu_{0.5}/TiO_2)_{OP}$, and $(Au_1Cu_1/TiO_2)_{OP}$ denote O_2 plasma treated (input power of 10 W and treatment time of 10 min) samples with the nominal Cu loadings of 0.2, 0.5, and 1 wt.%, respectively.

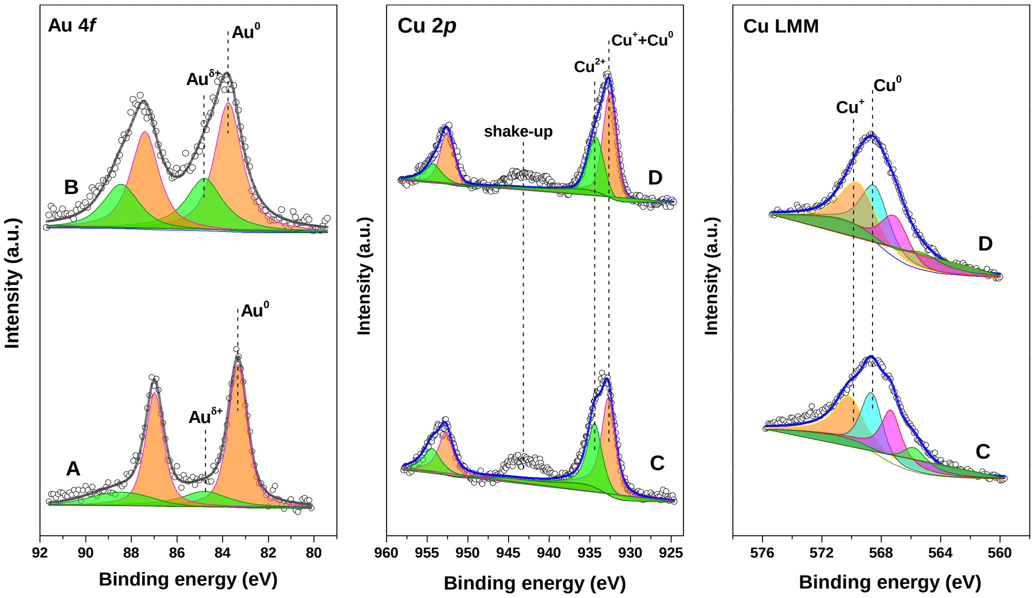

Figure S2 XPS Au 4*f* of the fresh (A) and used (B) $(Au_1/TiO_2)_{OP}$; XPS Cu 2*p* and Cu LMM of the fresh (C) and used (D) $(Cu_1/TiO_2)_{OP}$.

Table S1 XPS analysis of the plasmonic monometallic samples.

Sample	Au $4f_{7/2}$ (eV)		Cu $2p_{3/2}$ (eV)		Cu LMM (eV)		Au^0/Au (at.%)	Cu^{2+}/Cu (at.%)	Cu^0/Cu^+
	$Au^{\delta+}$	Au^0	Cu^{2+}	Cu	Cu^0	Cu^+			
A	84.9	83.7	-	-	-	-	58	-	-
B	84.7	83.4	-	-	-	-	80	-	-
C	-	-	934.3	932.7	568.7	570.2	-	41	0.97
D	-	-	934.5	932.5	568.5	570.0	-	39	0.72

Note: $Au=Au^0+Au^{\delta+}$; $Cu=Cu^0+Cu^++Cu^{2+}$; A and B denote the fresh and used $(Au_1/TiO_2)_{OP}$, respectively; C and D represent the fresh and used $(Cu_1/TiO_2)_{OP}$.

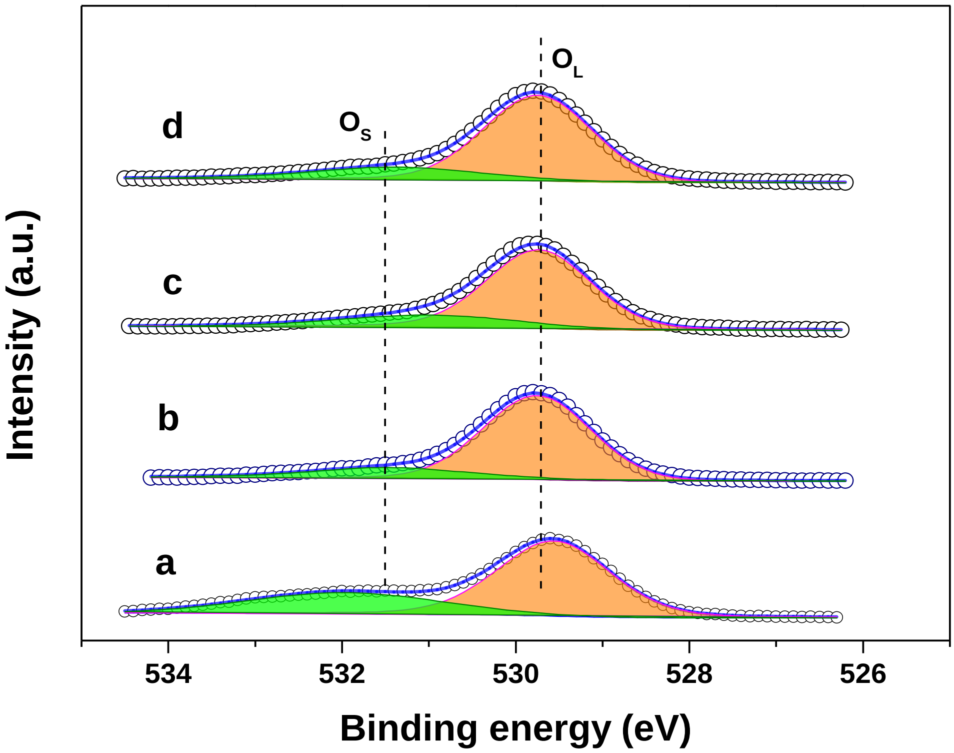

Figure S3 XPS O 1*s* of the fresh (a) and used (b) $(Au_1Cu_{0.5}/TiO_2)_{OP}$, and the fresh (c) and used (d) $(Au_1Cu_{0.5}/TiO_2)_{C200}$.

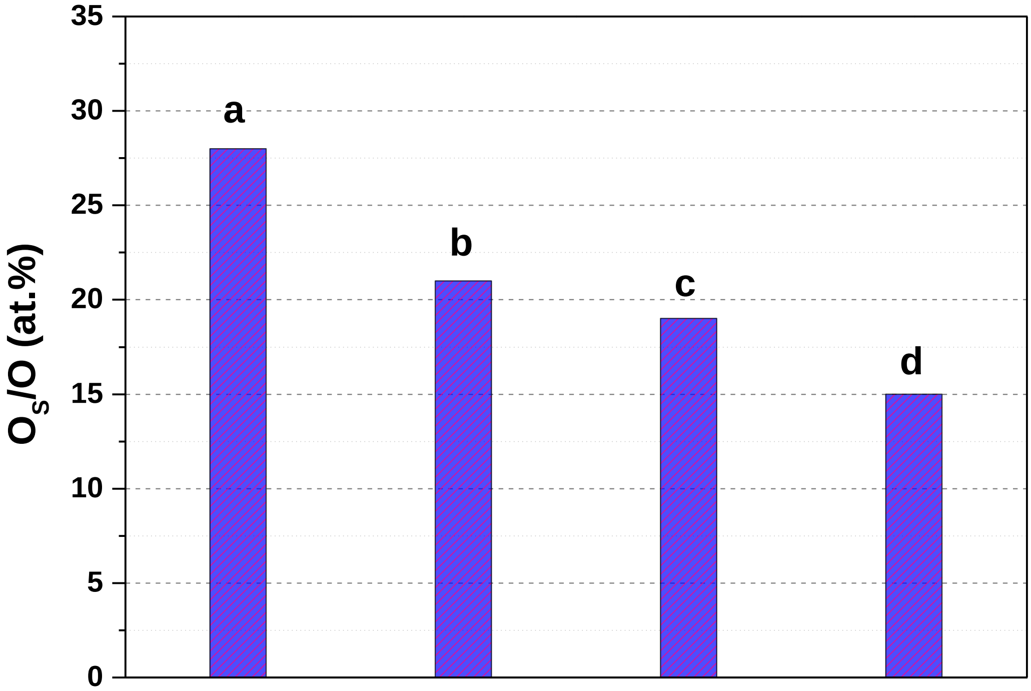

Figure S4 O_S contents of the fresh (a) and used (b) $(Au_1Cu_{0.5}/TiO_2)_{OP}$, and the fresh (c) and used (d) $(Au_1Cu_{0.5}/TiO_2)_{C200}$.

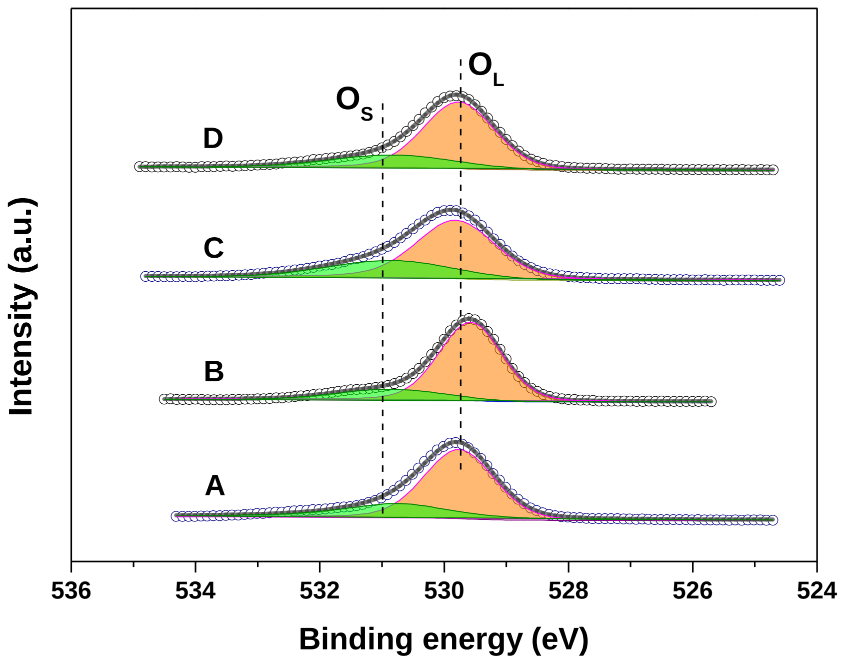

Figure S5 XPS O 1*s* of the fresh (A) and used (B) $(Au_1/TiO_2)_{OP}$, and the fresh (C) and used (D) $(Cu_1/TiO_2)_{OP}$.

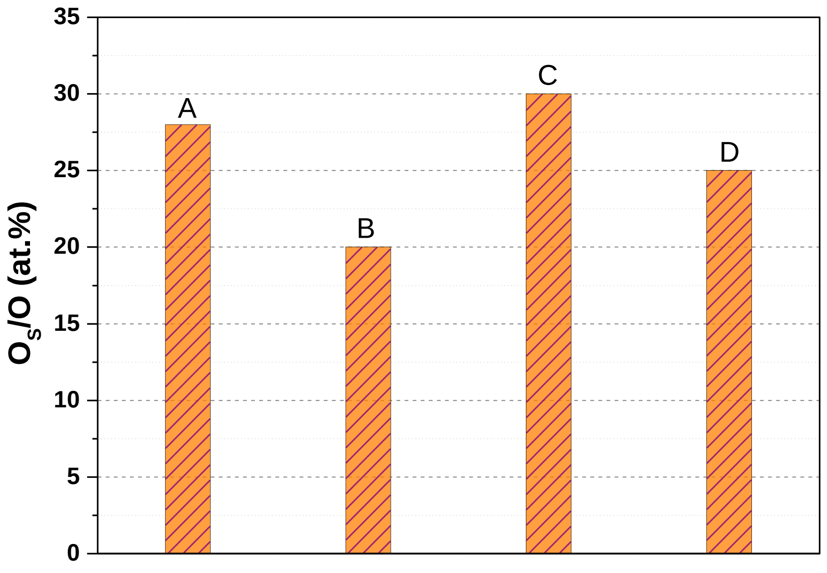

Figure S6 O_S contents of the fresh (A) and used (B) $(Au_1/TiO_2)_{OP}$, and the fresh (C) and used (D) $(Cu_1/TiO_2)_{OP}$.

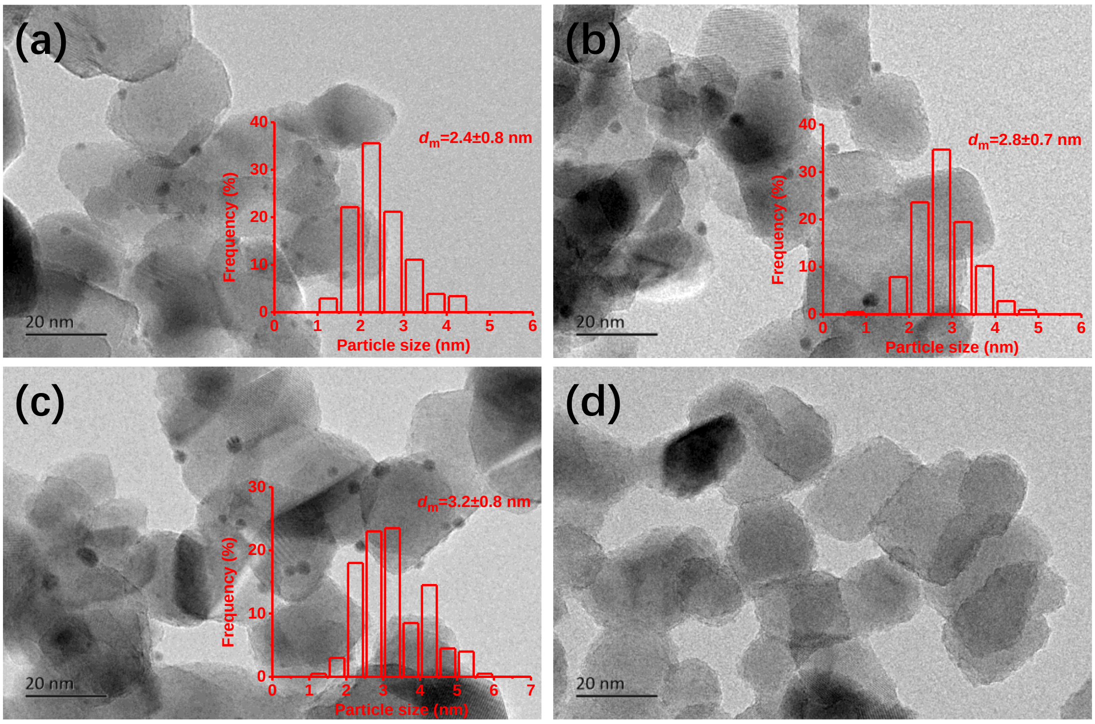

Figure S7 HR-TEM images and the particle size histograms of the used (a) $(Au_1Cu_{0.5}/TiO_2)_{OP}$, (b) $(Au_1Cu_{0.5}/TiO_2)_{C200}$, (c) $(Au_1/TiO_2)_{OP}$, (d) $(Cu_1/TiO_2)_{OP}$. The used samples were obtained after the PCO of CO for 90 min.

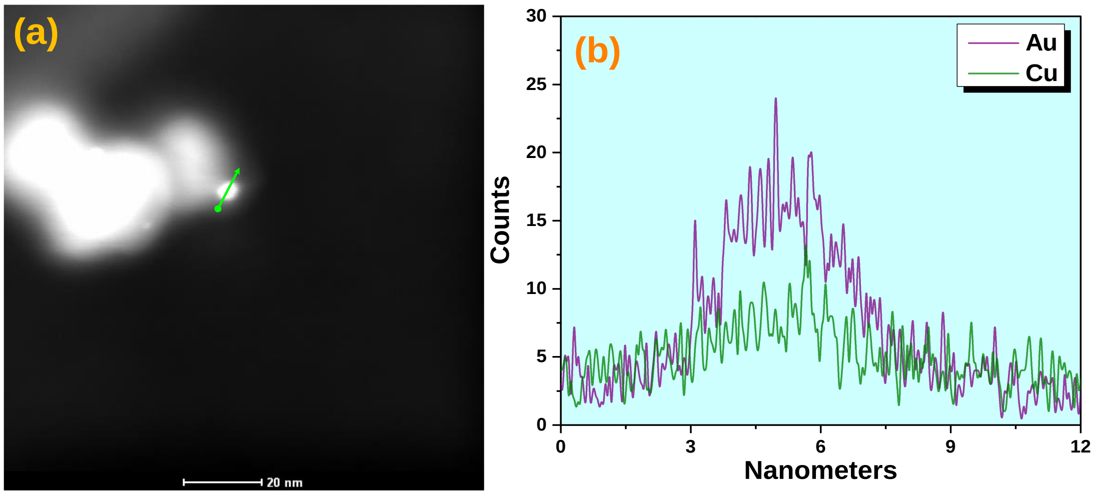

Figure S8 (a) HAADF-STEM image of the used $(Au_1Cu_{0.5}/TiO_2)_{OP}$, and (b) EDS line profiles for the plasmonic nanoparticle. The used nanocatalyst was obtained by applying the fresh sample to the VL PCO of CO for 90 min.

Estimation of the number of interfacial sites

To estimate the number of interfacial sites for the plasmonic nanoparticles, the Wulff equilibrium shape of nanoparticle was first constructed using VESTA software [1-3]. Then, we transformed the Wulff constructed nanoparticle (Figure S8) into physical models (see Figure S9) of the plasmonic nanoparticles for the $(Au_1Cu_{0.5}/TiO_2)_{OP}$, $(Au_1Cu_{0.5}/TiO_2)_{C200}$ and $(Au_1/TiO_2)_{OP}$ in Atomic Simulation Environment (ASE) based on the morphology and average size of the nanoparticles. Subsequently, the number of perimeter and total metal atoms for the physical models could be estimated, and the proportion of perimeter atoms can be calculated.

The construction of Wulff equilibrium shape depends on the surface energies, and the surface energies of Au and AuCu alloy were calculated by density functional theory (DFT). Briefly, the conventional cell of AuCu alloy was chosen from the Inorganic Crystal Structure Database (611749-ICSD). The Perdew-Burke-Ernzerhof (PBE) was employed in exchange-correlation functional, and the van der Waals (vdW) interaction was consideration. The plane-wave cut-off was set to be 400 eV, and 8×8×8 k-point grids were chosen in the geometry optimization of the bulk structure, whereas 9×9×1 k-point grids was selected for slab models. Periodic slabs were used to model low Miller index surface facets {100}, {110}, and {111} and higher index facets, {210}, {211}, {310}, and {311} [1]. A 20 Å vacuum gap was used to avoid any image-image interaction caused by the periodic boundary condition. The top two layers were fully relaxed while all other atoms were fixed. The surface energies, γ_{hkl}, were calculated as $\gamma_{hkl} = (E_{slab} - N{*}E_{bulk})/2A_{cell}$, where E_{slab} is the total energy of the slab, N is the number

of atoms in the slab, E_{bulk} is the energy of each atom in bulk, and A_{cell} is the surface area of unit cell.

Table S2 DFT calculated surface energies for different facets of Au and AuCu alloy, and their ratios with respect to the {111} facet surface energy.

Surface	γ_{hkl} (J/m^2)		Ratio to γ_{111}	
	Au	Au-Cu	Au	Au-Cu
{111}	1.37	1.74	1.00	1.00
{100}	1.52	1.88	1.11	1.08
{110}	1.60	2.02	1.17	1.16
{211}	1.58	1.92	1.15	1.10
{210}	1.59	2.02	1.16	1.16
{311}	1.58	1.97	1.15	1.13
{310}	1.60	2.09	1.17	1.20

Note: γ_{hkl} represents surface energy of the {hkl} facet

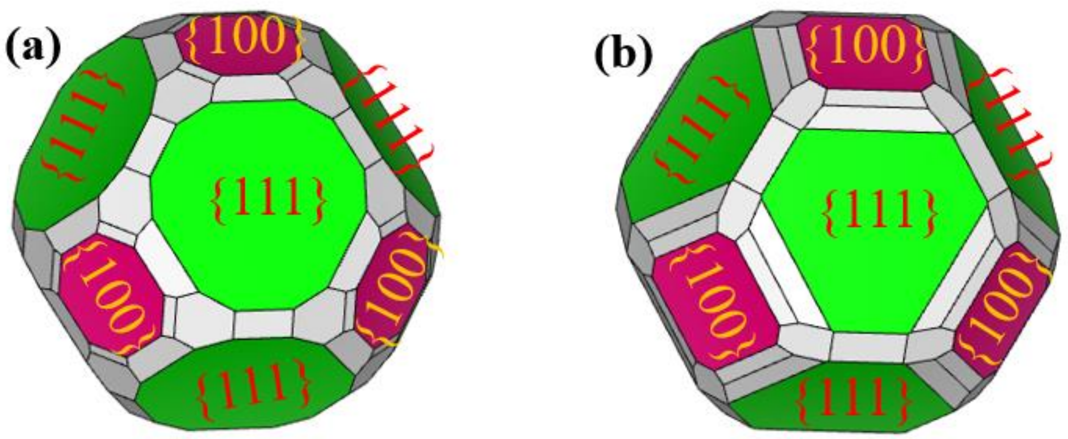

Figure S9 Wulff constructions of nanoparticle based on DFT calculated surface energies for (a) Au and (b) AuCu alloy.

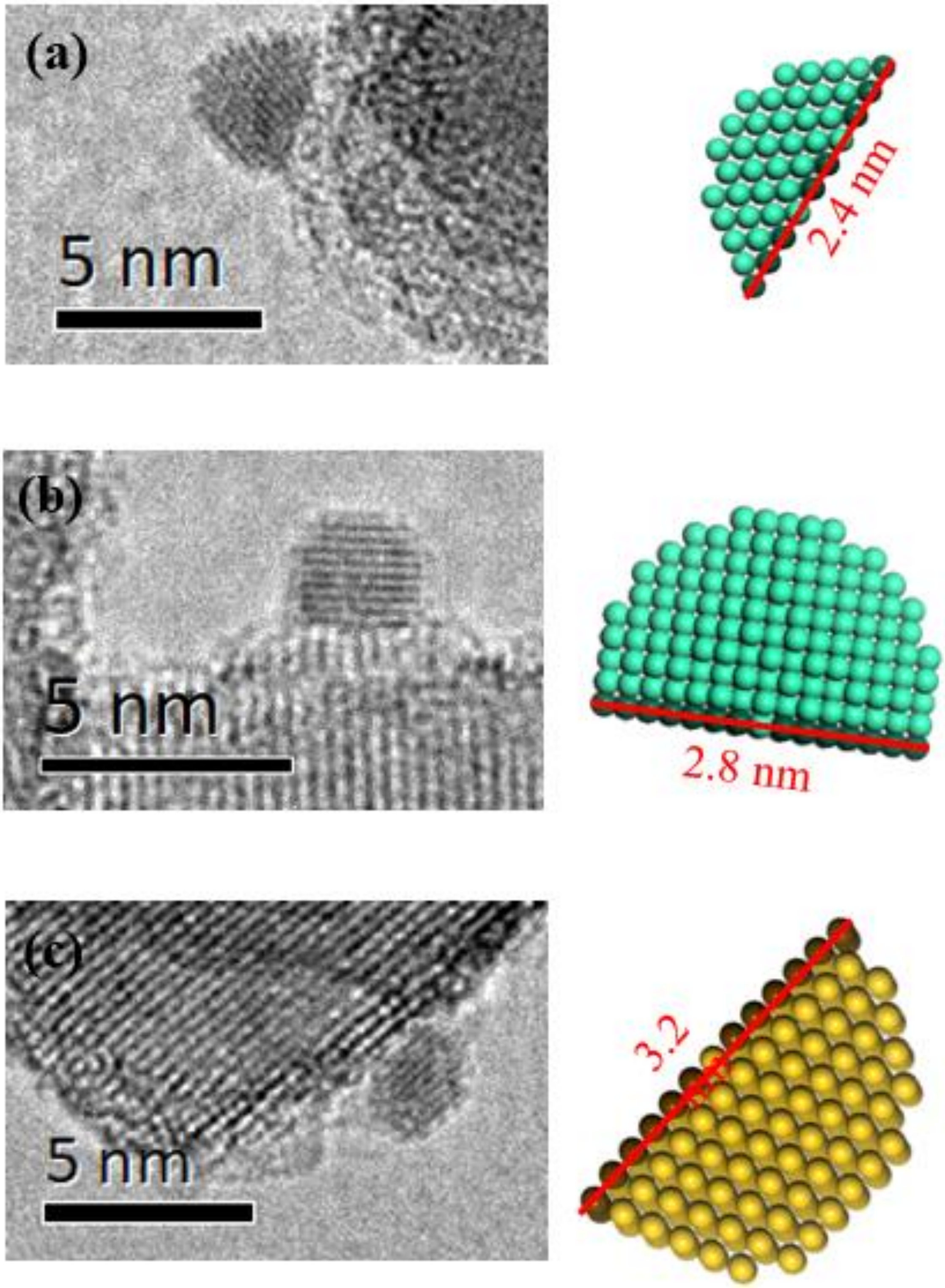

Figure S10 (Right) Physical models of perimeter sites (khaki ball) on truncated nanoparticles with 267, 601, and 564 atoms based on (left) HR-TEM images of the plasmonic nanoparticles for (a) $(Au_1Cu_{0.5}/TiO_2)_{OP}$, (b) $(Au_1Cu_{0.5}/TiO_2)_{C200}$ and (c) $(Au_1/TiO_2)_{OP}$. The used nanocatalysts were obtained by applying the fresh samples to the VL PCO of CO for 90 min.

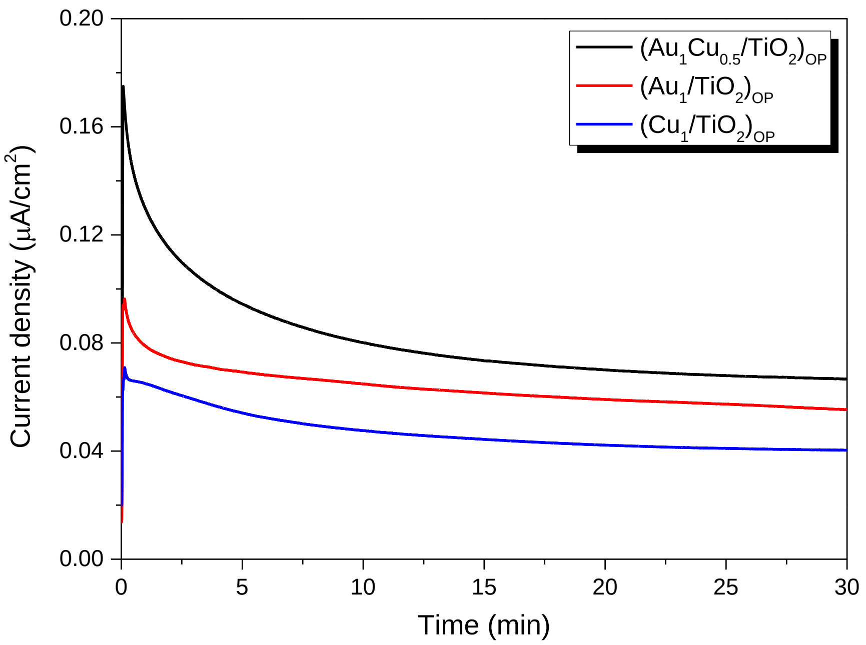

Figure S11 Photocurrent density for the used plasmonic nanocatalysts treated by O_2 plasma under VL irradiation (58 mW/cm^2; bias voltage of 0.5 V). The used nanocatalysts were obtained by applying the fresh samples to the VL PCO of CO for 90 min.

DFT calculation

CO oxidation calculations were investigated by VASP. To simulate the dissociation of O_2^- that formed from the combination of hot electron and O_2 molecules, NELECT and MAGMOM were adjusted. The Perdew-Burke-Ernzerhof (PBE) was employed in exchange-correlation functional, and spin polarization and van der Waals (vdW) interaction were consideration during the calculation. The plane-wave cut-off was set as 400 eV, and 8×8×8 k-point grids were chosen in the geometry optimization of the bulk structure, whereas Gamma k-point was selected for slab models. The transition states were found by employing a climbing image nudged elastic band (CI-NEB) method. The convergence threshold for the geometries optimized and CI-NEB were 0.03 eV/Å and 0.1 eV/Å, respectively [4,5]. The rutile (110) surface was modeled by a supercell (2 × 4). The oxide slab contained 5 layers, and a 15 Å vacuum gap was used [6]. The bottom two layers were fixed, and all other atoms were fully relaxed.

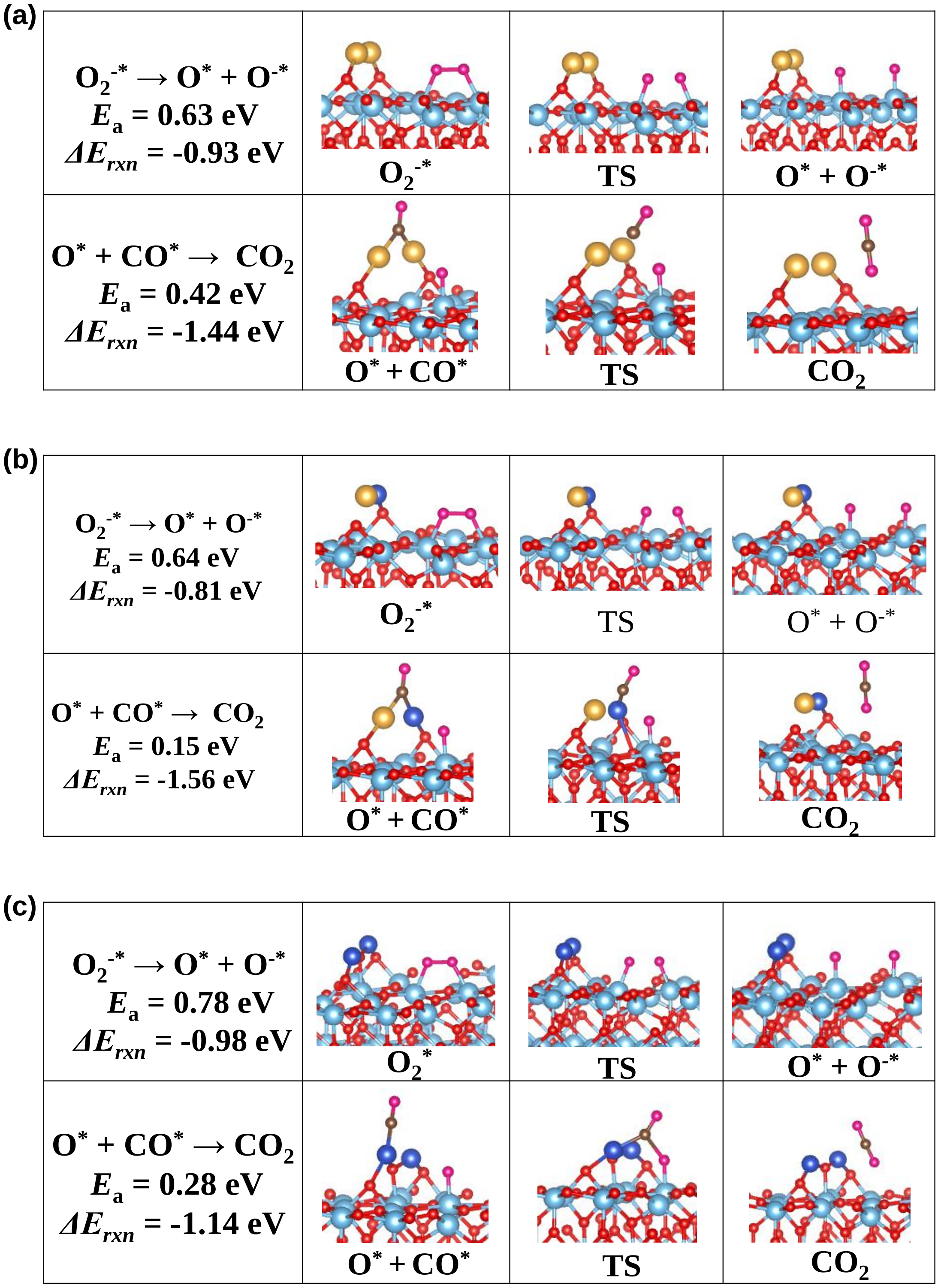

Figure S12 Optimized structures for the elementary steps of the whole CO oxidation pathway over (a) Au/TiO_2, (b) Au-Cu/TiO_2 and (c) CuO_x/TiO_2. Color code: Au, golden; Cu, blue; Ti, sky blue; C, brown; O atom in TiO_2, red; O atom in purple; O atom in CO, purple.

References

[1] T. Avanesian, S. Dai, J.M. Kale, W.G. Graham, X. Pan, P. Christopher. Quantitative and Atomic-Scale View of CO-Induced Pt Nanoparticle Surface Reconstruction at Saturation Coverage via DFT Calculations Coupled with in Situ TEM and IR, J. Am. Chem. Soc.139 (2017) 4551-4558.

[2] G.D. Barmparis, I.N. Remediakis, Dependence on CO adsorption of the shapes of multifaceted gold nanoparticles: a density functional theory, Rev. B: Condens. Matter Mater. Phys. 86 (2012) 085457.

[3] Y.C. Li, X.S. Li, B. Zhu, X. Zhu, H.Y. Lian, A.M. Zhu, A facile approach to direct preparation of Pt nanocatalysts from oxidative dechloridation of supported H_2PtCl_6 by oxygen plasma, J. Catal. 414 (2022) 16-24.

[4] T.T.H. Nguyen, V.K. Le, C.L. Minh, N.H. Nguyen, A theoretical study of carbon dioxide adsorption and activation on metal-doped (Fe, Co, Ni) carbon nanotube, Comput. Theor. Chem. 1100 (2017) 46-51.

[5] Liu, C. Lourenco, M. P. Hedstrom, S. Cavalca, F. Diaz-Morales, O. Duarte, H. A. Nilsson, A. Pettersson, L. G. M., Stability and Effects of Subsurface Oxygen in Oxide-Derived Cu Catalyst for CO_2 Reduction. J. Phys. Chem. C 121 (2017) 25010-25017.

[6] L.M. Liu, B. McAllister, H.Q. Ye, P. Hu, Identifying an O_2 Supply Pathway in CO Oxidation on Au/TiO_2 (110): A Density Functional Theory Study on the Intrinsic Role of Water, J. Am. Chem. Soc.128 (2006) 4017-4022.
